# Supplementary material for: Atomic-Scale Imaging of Transferred Graphene Nanoribbons for Nanoelectronic Device Integration
Source: ACS Appl Nano Mater. 2025 Aug 12;8(33):16457–64. doi: 10.1021/acsanm.5c02753 (PMC12379158; doi:10.1021/acsanm.5c02753)
Supplement: Supplementary file 1 [file an5c02753_si_001.pdf]

Supporting Information

Atomic-Scale Imaging of Transferred Graphene Nanoribbons for Nanoelectronic Device Integration

Amogh Kinikar[a], Feifei Xiang[a], Lucia Palomino-Ruiz[a,b+], Li-Syuan Lu[c], Chengye Dong[d], Yanwei Gu[e#], Rimah Darawish[a,f], Eve Ammerman[a], Oliver Gröning[a], Klaus Müllen[e,g], Roman Fasel[a,f], Joshua A. Robinson[c,d,h], Pascal Ruffieux[a], Bruno Schuler[a], Gabriela Borin Barin[a*]

[a]nanotech@surfaces laboratory, Empa - Swiss Federal Laboratories for Materials Science and Technology, 8600 Dübendorf, Switzerland.

[b]Departamento de Química Orgánica, Facultad de Ciencias, Unidad de Excelencia de Química Aplicada a Biomedicina y Medioambiente (UEQ), Universidad de Granada, 18071 Granada, Spain

[c]Department of Materials Science and Engineering, The Pennsylvania State University, University Park, Pennsylvania 16082, USA

[d]Two-Dimensional Crystal Consortium, The Pennsylvania State University, University Park, Pennsylvania 16802, USA

[e]Max Planck Institute for Polymer Research, 55128 Mainz, Germany

[f]Department of Chemistry, Biochemistry and Pharmaceutical Sciences, University of Bern, 3012 Bern, Switzerland

[g]Department of Chemistry, Johannes Gutenberg University Mainz, Duesbergweg 10-14, 55128, Mainz, Germany

[h]Department of Chemistry and Department of Physics, The Pennsylvania State University, University Park, Pennsylvania 16802, USA

[+]Current affiliation: Fundación IMDEA Nanociencia, 28049, Madrid, Spain

[#]Current affiliation: Ningbo Institute of Materials Technology & Engineering, Chinese Academy of Sciences, Ningbo, 315201, P.R. China

corresponding author: gabriela.borin-barin@empa.ch

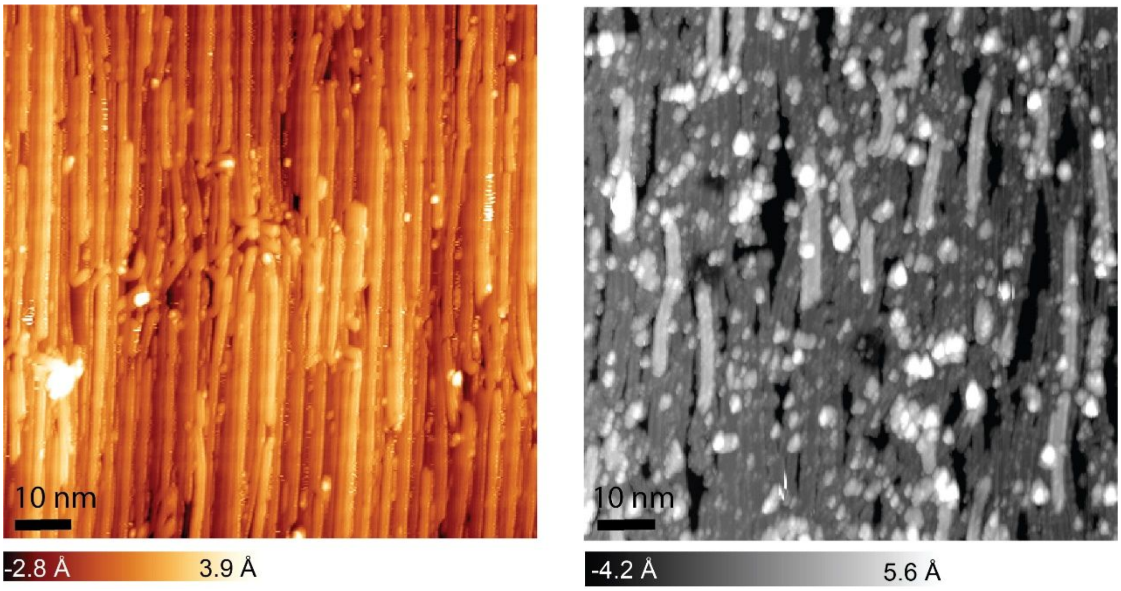

Figure S1. (left panel) STM image of aligned 9-AGNRs as synthesized on Au(788) (-1.5 V, 30 pA). (right panel) STM image of aligned 9-AGNRs transferred on EG (-4.6 V, 10 pA).

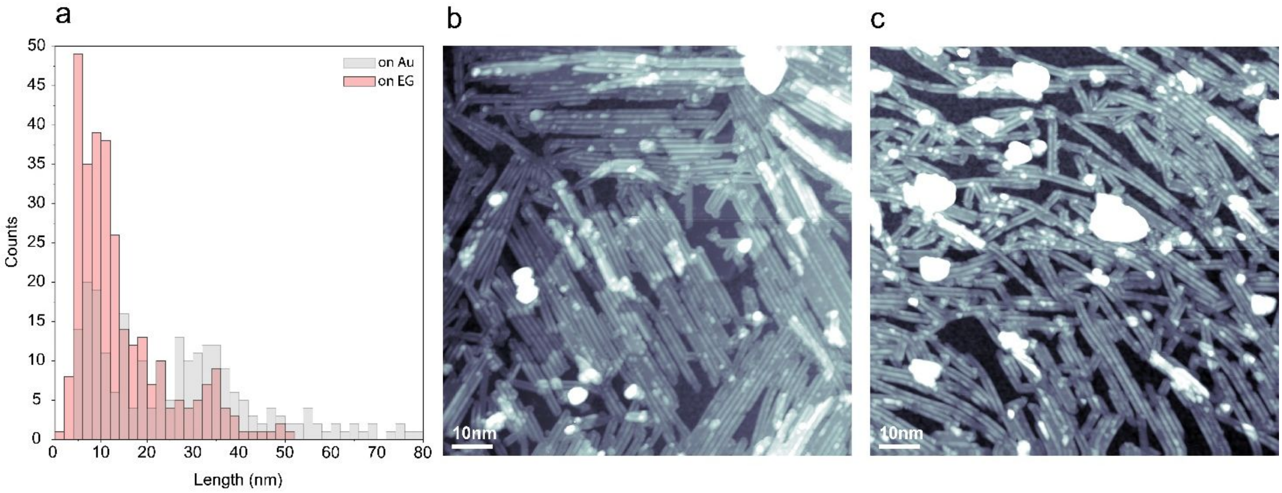

Figure S2. a) GNR length distribution before transfer on Au (light grey; figure 1b as a representative image) and after transfer on EG (pink), b-c. Representative 9-AGNR images on EG were used for quantifying GNR length. *Images on EG are challenging for statistical analysis, which could lead to underestimating the number of longer ribbons on EG.

The observed reduction in GNR length from an average of 26 nm pre-transfer to 15 nm post-transfer is primarily attributed to mechanical/chemical fragmentation during wet transfer and thermal fragmentation during UHV annealing. Capillary forces and interfacial stresses introduced during delamination, etching, and liquid-based processing likely induce mechanical strain, leading to breakage at intrinsic defect sites. Additionally, the high-temperature UHV annealing step, necessary for impurity desorption, may further contribute to length reduction by inducing thermally driven cleavage at pre-existing weak points in the GNRs. The presence of some fused GNRs suggests that, while annealing promotes impurity removal, it also leads to limited inter-GNR interactions

that may influence their final morphology. The absence of oxidation-related modifications in STM and Raman spectra indicates that chemical degradation during gold etching is not a dominant factor. These findings highlight the importance of optimizing transfer and annealing conditions to mitigate structural fragmentation and improve GNR quality for device applications.

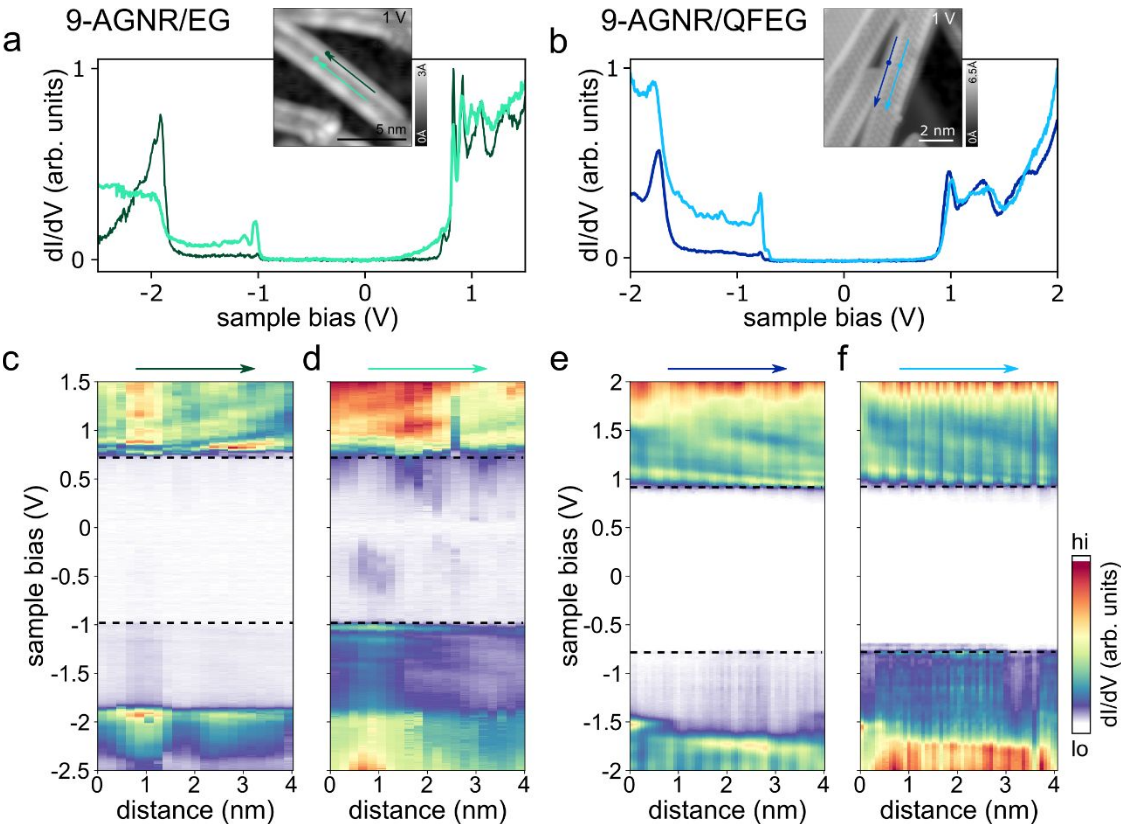

Figure S3. STS along 9-AGNR on EG and QFEG. **a** dI/dV spectra of 9-AGNR on EG at inner (light green) and outer (dark green) ribbon positions (see points in inset). **b** dI/dV spectra of 9-AGNR on QFEG at inner (light blue) and outer (dark blue) ribbon positions (see points in inset). **c,d** dI/dV spectra along the outer (c) and inner (d) part of 9-AGNR on EG (see dark and light green arrow in inset in a). **e,f** dI/dV spectra along the outer (e) and inner (f) part of 9-AGNR on QFEG (see dark and light blue arrow in inset in b).

CoPor-DBA synthesis

All the reactions were performed in Argon atmosphere supplied into flasks via manifolds. Solvents and reagents were purchased from TCI, Sigma-Aldrich, Acros, Merck, and other commercial suppliers and used without further purification unless otherwise noted. Anhydrous tetrahydrofuran, dichloromethane, and dimethyl sulfoxide were purchased from Sigma-Aldrich and Acros. Column chromatography was conducted with silica gel (grain size 0.063–0.200 mm or 0.04–0.063 mm) and thin-layer chromatography (TLC) was performed on silica gel-coated aluminum sheets with F254 indicator. The high-resolution time-of-flight mass spectrometry (MALDI-TOF) measurements have been performed on a SYNAPT G2 Si high resolution time-of-flight mass

spectrometer (Waters Corp., Manchester, UK) with matrix-assisted laser desorption/ionization (MALDI) source. X-ray crystallographic data for precursors **CoPor-DBA** was collected on an IPDS 2T diffractometer using a STOE IPDS 2T diffractometer with a Mo–Kα IμS mirror system radiation. The structure was solved by direct methods SIR-2004 and refined by SHELXL-2014 (full matrix), 450 refined parameters.

Synthetic procedures

Cobalt (II) 5-(2,7-dibromoanthracen-9-yl)porphyrin (**CoPor-DBA**)

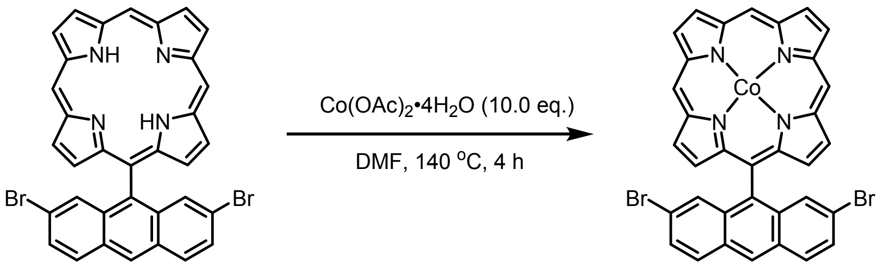

2HPor-DBA

CoPor-DBA
89%

In a 100 mL round bottom flask, cobalt acetate tetrahydrate (124.5 mg, 0.5 mmol, 10.0 equiv.) was added to a solution of compound **2HPor-DBA** (32.0 mg, 0.05 mmol) in *N,N*-Dimethylformamide (30 mL), and the mixture was stirred for 4 hours at 140 ℃. The solvent was removed under vacuum and the residue was purified by silica gel column chromatography (hexane/dichloromethane =7/3) to afford the desired product **CoPor-DBA** (31.2 mg, 89% yield) as pink solid. The further purified compound **CoPor-DBA** for on-surface synthesis was achieved by solvent wash with methanol. Due to its paramagnetic ground state, the proton and carbon nmr were not collected. HRMS (MALDI-TOF, positive) *m*/*z*: $[M]^+$ calcd for $C_{34}H_{18}Br_2N_4Co$, 698.9230; found: 698.9201. The molecular structure was further confirmed by the single crystal X-ray crystallography.

Single crystal X-ray diffraction analysis

The single crystal of precursor **CoPor-DBA** suitable for X-ray analysis was obtained by slow diffusion of methanol into the solution of **CoPor-DBA** in dichloromethane with the exclusion of light. Crystallographic data for compound **CoPor-DBA** is available free of charge from the Cambridge Crystallographic Data Centre under CCDC identifiers 2431713 (www.ccdc.cam.ac.uk/structures/).

Table S1. Crystal data and structure refinement for **CoPor-DBA**.

Moiety formula	$C_{34}H_{18}Br_2CoN_4$
Formula weight	701.27 g/mol
Temperature	120(2) K

Wavelength	0.71073Å, MoKα
Diffractometer	STOE IPDS 2T
Crystal system	Orthorhombic
Space group	P bca, (61)
Unit cell dimensions	a = 9.6657(7) Å
	b = 14.5808(12) Å
	c = 37.059(4) Å
Volume	5222.9(8) $Å^3$
Number of reflections	5787
and range used for lattice parameters	2.38° <= θ <= 28.17°
Z	8
Density (calculated)	1.784 Mg/m^3
Absorption coefficient	3.751 mm^{-1}
Absorption correction	Integration
Max. and min. transmission	0.8681 and 0.1832
F(000)	2776
Crystal size, colour and form	0.040 × 0.070 × 0.760 mm^3, brown needle
Theta range for data collection	2.377 to 28.063°.
Index ranges	-10<=h<=12, -19<=k<=16, -41<=l<=48
Reflections collected	14927

Independent reflections	6235 [R(int) = 0.1771]
observed [I>2sigma(I)]	2133
Completeness to theta = 67.7°	99.8 %
Refinement method	Full-matrix least-squares on F^2
Data /restraints / parameters	6235 / 0 / 370
Goodness-of-fit on F^2	1.045
Final R indices [I>2sigma(I)]	R1 = 0.1257, wR2 = 0.2688
R indices (all data)	R1 = 0.3033, wR2 = 0.3856
Largest diff. peak and hole	1.112 and -0.773 eÅ^{-3}

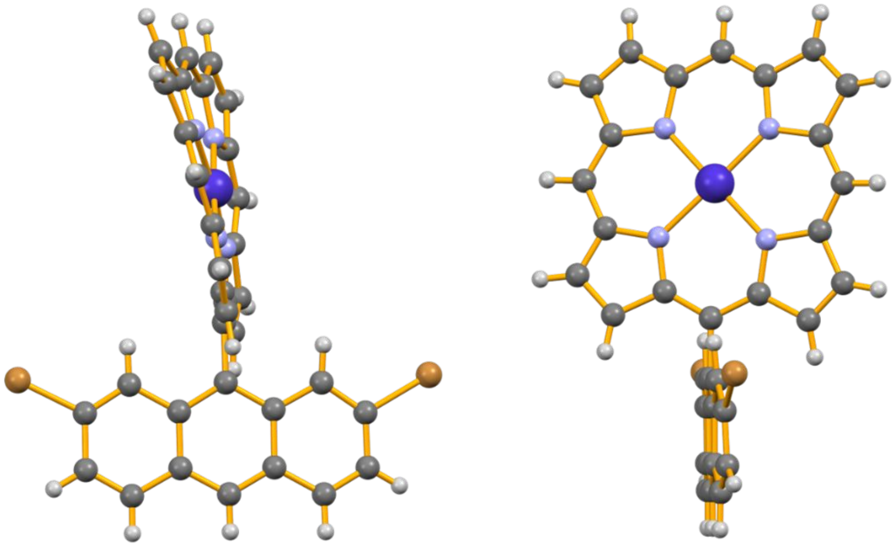

Figure S4. X-ray single-crystal analysis of **CoPor-DBA**. Bromine, nitrogen, and cobalt atoms are labelled in brown, purple and blue.

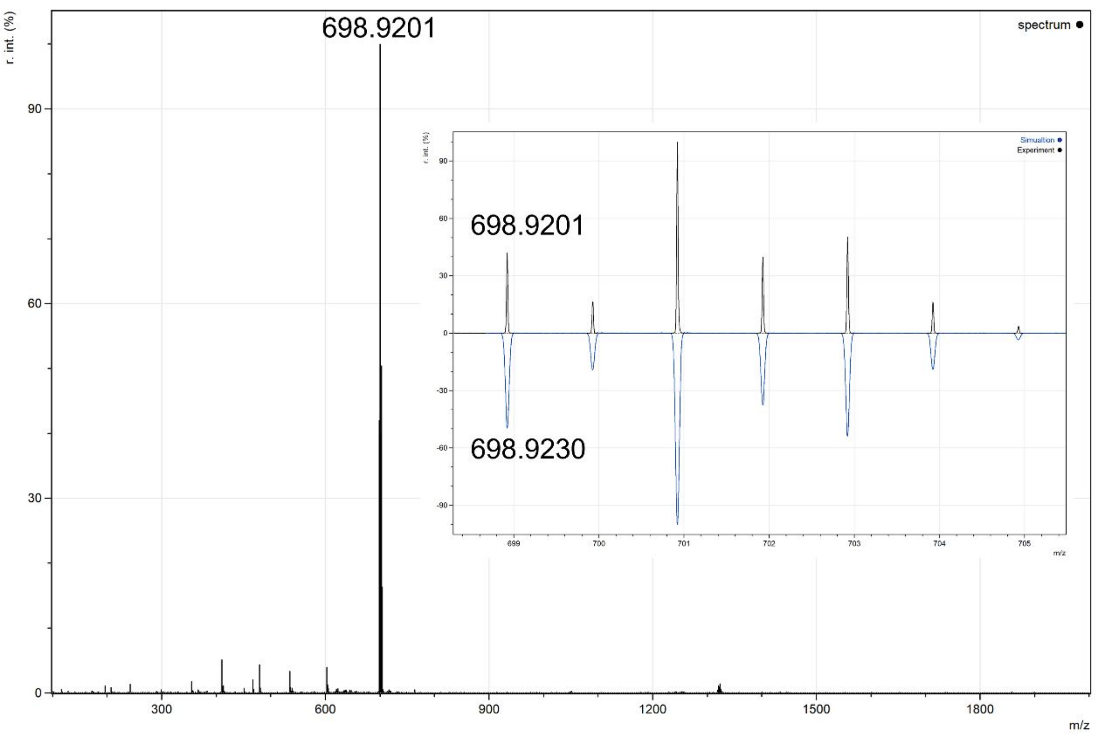

Figure S5. High-resolution MALDI-TOF mass spectrum of precursor CoPor-DBA. Inset displays the isotopic distribution (black colour) in comparison to the simulated pattern (blue colour).
